# Cytotoxic and apoptotic evaluations of marine bacteria isolated from brine-seawater interface of the Red Sea

**DOI:** 10.1186/1472-6882-13-29

**Published:** 2013-02-06

**Authors:** Sunil Sagar, Luke Esau, Tyas Hikmawan, Andre Antunes, Karie Holtermann, Ulrich Stingl, Vladimir B Bajic, Mandeep Kaur

**Affiliations:** 1 King Abdullah University of Science and Technology (KAUST), Computational Bioscience Research Center, Thuwal, 23955-6900, Kingdom of Saudi Arabia; 2 King Abdullah University of Science and Technology (KAUST), Red Sea Research Center, Thuwal, 23955-6900, Kingdom of Saudi Arabia

**Keywords:** Marine bacteria, Deep sea brine pools, Extracts, Cytotoxicity, Apoptosis

## Abstract

**Background:**

High salinity and temperature combined with presence of heavy metals and low oxygen renders deep-sea anoxic brines of the Red Sea as one of the most extreme environments on Earth. The ability to adapt and survive in these extreme environments makes inhabiting bacteria interesting candidates for the search of novel bioactive molecules.

**Methods:**

Total 20 i.e. lipophilic (chloroform) and hydrophilic (70% ethanol) extracts of marine bacteria isolated from brine-seawater interface of the Red Sea were tested for cytotoxic and apoptotic activity against three human cancer cell lines, i.e. HeLa (cervical carcinoma), MCF-7 (Breast Adenocarcinoma) and DU145 (Prostate carcinoma).

**Results:**

Among these, twelve extracts were found to be very active after 24 hours of treatment, which were further evaluated for their cytotoxic and apoptotic effects at 48 hr. The extracts from the isolates P1-37B and P3-37A (*Halomonas*) and P1-17B (*Sulfitobacter*) have been found to be the most potent against tested cancer cell lines.

**Conclusion:**

Overall, bacterial isolates from the Red Sea displayed promising results and can be explored further to find novel drug-like molecules. The cell line specific activity of the extracts may be attributed to the presence of different polarity compounds or the cancer type i.e. biological differences in cell lines and different mechanisms of action of programmed cell death prevalent in different cancer cell lines.

## Background

The marine environment is a rich source of unique bioactive molecules with unlimited chemical and functional diversity. Approximately 30,000 natural products have been isolated from marine organisms (http://dmnp.chemnetbase.com/intro/index.jsp) and many of the drug candidates are currently in clinical trials [1,2]. In the recent reports, both, deep sea water bodies and seafloors have been shown to be some of the most biodiverse and species-rich habitats on the planet [3,4]. The continuous search for new chemically diverse molecules and the development of new methods for culturing deep-sea microorganisms have placed the deep sea environment as a new frontier in the drug discovery process. The general opinion is that the survival of deep sea organisms in the extreme conditions of pressure, absence of light, low levels of oxygen, high temperature and salt concentration may have affect their primary and secondary metabolite profiles [5,6] which, in turn, can be suitable candidates for discovery of structurally unique molecules.

Siderophore Bisucaberin [7] was the first cytotoxic compound isolated from deep-sea marine bacteria *Alteromonas haloplanktis*, collected at 3000 m depth off the coast of Aomori Prefecture, Japan. Lately, Homann et al. reported the isolation of peptidic siderophores Loihichelins A-F [8] from the cultures of heterotrophic bacterium *Halomonas* LOB-5 collected from the southern rift zone of Loihi Seamount east of Hawaii. Novel cytotoxic phenazine derivatives [9] with unique ring system have been described from pacific sediment *Bacillus* sp. (5059 m depth). Several other compounds with significant cytotoxic activity which were isolated from deep-sea *Bacillus* and *Streptomyces* sp. and actinomycete have been described by Danielle Skropeta in a recent review [10]. In our effort to exploit the biodiversity of these environments, several strains of marine bacteria were isolated from Kebrit Deep, Nereus Deep, Discovery Deep and Erba Deep of the Red Sea [11] and evaluated for their cytotoxic and apoptotic potential.

The Red Sea harbors approximately 25 deep-sea anoxic brine pools, which were formed by a process of re-dissolution of evaporitic deposits [12-14]. The deep-sea anoxic brines of the Red Sea are considered to be one of the most extreme environments on Earth, which are characterized by drastic changes in physicochemical conditions when compared to overlying seawater. This includes the increases in salinity (from 4% up to 26% salinity), temperature (up to 70°C), concentrations of heavy metals, and decrease in oxygen [15,16].

The present study describes the assessment of anticancer activity of marine microorganisms from the brine-seawater interface of the Red Sea. The cytotoxic activities of lipophilic, i.e. chloroform and hydrophilic, i.e. 70% ethanol extracts of marine bacteria isolated from deep sea brine pools of Red Sea, against HeLa (cervical carcinoma), MCF-7 (breast adenocarcinoma) and DU145 (prostate carcinoma) cell lines, were measured at 24 hr time point. Some of the highly active extracts were further assessed for cytotoxicity and apoptotic activity at 48 hr.

## Methods

### Field sampling

Water samples from the brine-seawater interface were collected using a rosette sampler equipped with 10 l Niskin bottles and a conductivity-temperature-depths (CTD) unit for monitoring salinity, temperature, transmission, and pressure. At each sampling site, 180 l of samples were collected and pre-filtered through a 5.0 μm SMWP membrane (diameter 290 mm; Millipore, Ireland) to remove suspended particles, then filter-concentrated with Tangential Flow Filtration (TFF) system through Durapore 0.1 μm PVDF filter (Pellicon 2 Cassette Filter, Screen type C, size 0.5 m^2^, Millipore Corporation, MA, USA). After filtration, one liter of each concentrated sample was stored in the dark at 4°C and was used as inoculum.

### Source of bacterial isolates

Twelve strains of bacteria investigated in this study were isolated from brine-seawater interface of deep sea brines in the Red Sea. Brine-seawater samples for bacterial isolation were collected from four different deep brines named Discovery Deep (2050 m), Kebrit Deep (1549 m), Nereus Deep (2300 m), and Erba Deep (2300 m). Each of the deep sea brine has its unique physicochemical composition [11]. In the Discovery deep, brine–seawater boundaries are characterized by strong temperature and salinity gradients [14].

All of the bacterial strains isolated in this study were obtained by the streak plate method described elsewhere [17]. All of the strains grew in 10% NaCl with the exception of one strain isolated from Discovery Deep. This strain was successfully isolated using 20% NaCl.

### PCR amplification

PCR amplifications of the extracted DNA of the cultures were performed in a 25 μl reaction having 12.5 μl Promega PCR Master Mix (Promega, USA), 0.5 μl (final concentration 0.5 μM) of primer 27bF (5’-AGAGTTTGATCMTGGCTCAG-3’) and 1492uR (5’-TACCTTGTTACGACTT-3’). PCR reaction were carried out in Mastercycler (Eppendorf, Germany) under the following conditions: a pre-denaturation temperature of 95°C for 5 min; 30 cycles of 95°C for 60s, 55°C for 60s, 72°C for 30s; followed by final extension at 72°C for 5 min. The yield and quality of the PCR products were examined on 1% (wt/vol) agarose gel (SeaKem GTG, Lonza, USA) stained with SYBR Safe (Invitrogen, USA), then purified with Illustra Exostar 1-step (GE, Healthcare, UK) according to the manufacturer’s protocol. The 16S rRNA genes were sequenced with an ABI 3730xl capillary DNA sequencer (PE Applied Biosystems), at Core Lab. KAUST, Saudi Arabia.

### Bacterial biomass

Strains of halophilic bacteria were isolated into pure culture by streaking the samples onto three different solid media types, marine agar 2216 (Difco), plate count agar (Teknova), and R2A agar (Oxoid). These media were supplemented with either 10% or 20% NaCl to adjust salinity. All plates were incubated at 30°C and inspected daily for two weeks. Colonies with different morphologies were picked and pure cultures of these colonies were obtained after three transfers. Taxonomic identifications were based on 16S rRNA gene sequencing. DNA extraction of the pure cultures, 16S rRNA gene amplification, and sequencing were performed according to [18]. A BLASTN search was used to identify strains to their closest relatives in GenBank. To prepare extracts, bacteria were grown in 1 L of Marine Broth (Difco) supplemented with NaCl. Cultures were incubated at 30°C in a shaking incubator and harvested after two weeks by centrifugation. Cell pellets were frozen at **−**80°C until used for extract preparation.

### Extract preparation

Lipophilic, i.e. chloroform and hydrophilic, i.e. 70% ethanol, extracts of 12 strains of marine bacteria were prepared at a concentration of 100 mg mL^-1^ (speedvac dried material/mL of solvent). Solutions were sonicated with ultra-sound probe (Biologics Inc., Model 150 V/T) for 3 × 2 minutes on ice. The solutions were centrifuged at 10000 g for 15 minutes; the supernatants were recovered and stored at −20°C.

### Cell culture

MCF-7 (Breast Adenocarcinoma), DU145 (Prostate carcinoma) and HeLa (Cervical carcinoma) were obtained from the American Type Cell Culture Collection (ATCC, Manassas, VA). All cell lines were cultured in DMEM (Dulbecco’s Modified Eagle’s Medium), supplemented with 10% FCS (Fetal calf serum), penicillin (100U/ml) and streptomycin (100 μg/ml) at 5% CO_2_ in a 37°C incubator.

### MTT assay

The cytotoxicity of marine bacterial extracts was estimated by MTT (3-(4, 5-Dimethylthiazol-2-yl)-2, 5-diphenyltetrazolium bromide) assay. Cells were seeded at a density of 2.5 × 10^3^ cells per well in a 384-well culture plates and treated with 1 mg/mL marine bacterial extract for 24 and 48 hr (hours). Cells were treated with 5 mM H_2_O_2_ as a positive control. Following incubation with extracts, 5 μl of sterile MTT (5 mg/mL) dissolved in PBS was added to each well and incubated with cells for 4 hr followed by the addition of 30 μl of solubilisation solution (10% SDS, 10 mM HCl) which was incubated with cells for 16 hr at 37°C. The OD (optical density) of each well was measured at 595 nm using a microtiter plate reader (BMG Labtech PHERAstar *FS*, Germany) and results were analyzed using Microsoft Office Excel^**©**^.

### APOPercentage assay

The cells were seeded in 96 well plates at a density of 5 × 10^3^ cells per well in duplicate in 90 μl of media. After 24 hr, cells were treated with marine bacterial extracts diluted in complete DMEM to a final concentration of 1 mg/mL and incubated at 37°C for 48 hr. Cells were treated with 2.5 mM H_2_O_2_ for 30 minutes as a positive control. The cells were stained with APOPercentage dye as per manufacturer’s instructions (Biocolor, UK). Duplicates were pooled, the OD was measured at 550 nm absorbance using a microtiter plate reader (BMG Labtech PHERAstar *FS*, Germany) and percentage staining was calculated.

### Statistical analysis

Student’s *t*-test was used to compare the samples (treated vs. Untreated) and were noted to be statistically significantly different where p<0.05. All statistics including mean and SD calculations were performed using Microsoft Office Excel^**©**^.

## Results

### Microbial isolates

Twelve strains of marine bacteria were isolated from the Kebrit Deep, Nereus Deep, Discovery Deep and Erba Deep of the Red Sea (Table 1). The isolation of bacteria and their culturing were done by Red Sea Research Center at King Abdullah University of Science and Technology. Total 20 extracts i.e. lipophilic (chloroform) and hydrophilic (70% ethanol) have been investigated for their anticancer potential against above mentioned three human cancer cell lines.

**Table 1:** Taxonomic identification, location of collection and source of origin for 12 microbial strains

**Chloroform extract**	**Ethanol extract**	**Isolate**	**Source**	**Closest relative**	**Similarity**	**GenBank access no.**	**Ref**
-	1E	P1-17B	Kebrit interface	*Sulfitobacter pontiacus*	99%	NR_026418.1	[19]
2C	-	P1-37B	Nereus interface	*Halomonas meridiana*	100%	AF212217.2	[20]
3C	3E	P3-82	Erba interface	*Halomonas meridiana*	100%	AF212217.2	[20]
4C	4E	P5-16A	Kebrit interface	*Halomonas axialensis*	100%	NR_027219.1	[21]
5C	5E	P5-16B	Kebrit interface	*Halomonas axialensis*	100%	NR_027219.1	[21]
6C	6E	P5-37A	Nereus interface	*Halomonas meridiana*	99%	AF212217.2	[20]
7C	7E	P5-82	Erba interface	*Idiomarina baltica*	99%	AJ440215.1	[22]
8C	-	P1-37A	Nereus interface	*Marinobacter bryozoorum*	98%	NR_027192.1	[23]
9C	9E	P1-82	Erba interface	*Halomonas meridiana*	100%	NR_042066.1	[24]
10C	10E	P1-16B	Kebrit interface	*Vibrio sinaloensis*	98%	NR_043858.1	[25]
11C	11E	P3-37A	Nereus interface	*Halomonas aquamarina*	100%	HQ188659.1	[26]
12C	-	P6-86	Discovery interface	*Chromohaloba-cter salexigens*	99%	EU868854.1	[27]

### Cytotoxic activities of bacterial extracts

**Figure 1:**
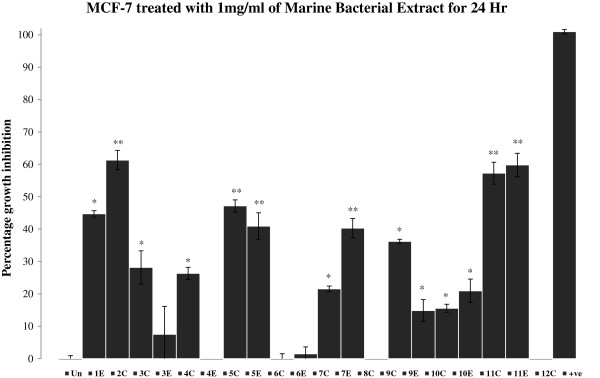
**Percent growth inhibition of MCF-7 cells after treatment with 1 mg/mL marine bacterial extracts for 24 hr.** Data were normalized to DMSO treatment controls, and error bars represent the standard deviation for triplicate experiments. Values marked as asterisk (*p<0.05 and **p<0.01) are significantly different from untreated control (Un). +ve represents positive control (H_2_O_2_).

**Figure 2:**
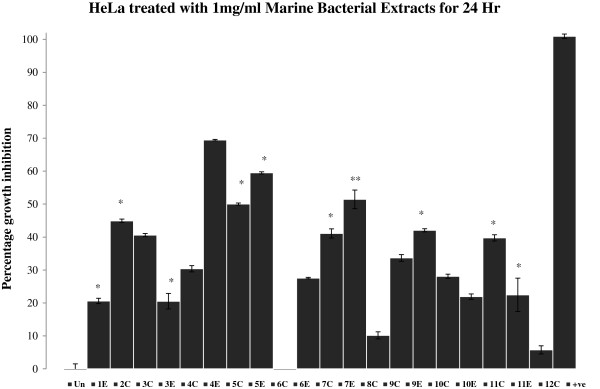
**Percent growth inhibition of HeLa cells after treatment with 1 mg/mL marine bacterial extracts for 24 hr.** Data were normalized to DMSO treatment controls, and error bars represent the standard deviation for triplicate experiments. Values marked as asterisk (*p<0.05 and **p<0.01) are significantly different from untreated control (Un). +ve represents positive control (H_2_O_2_).

**Figure 3:**
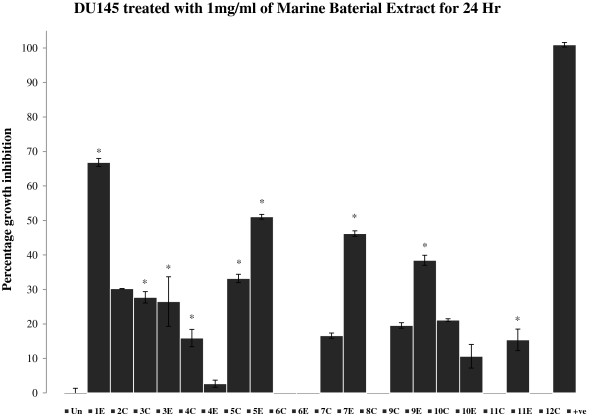
**Percent growth inhibition of DU145 cells after treatment with 1 mg/mL marine bacterial extracts for 24 hr.** Data were normalized to DMSO treatment controls, and error bars represent the standard deviation for triplicate experiments. Values marked as asterisk (*p<0.05 and **p<0.01) are significantly different from untreated control (Un). +ve represents positive control (H_2_O_2_).

Cytotoxic activities for the extracts were assessed by MTT assay. The growth inhibitions were measured after treating the cell lines with microbial extracts for 24 hr (Figures 1, 2 and 3). Out of total 20 bacterial extracts, 13 extracts i.e. **2C**, **5C**, **7C, 8C, 11C, 1E, 4E, 5E, 6E, 7E, 9E, 10E** and **11E** displayed maximum growth inhibitory effects on different cancer cell lines. The specific activity of different extracts for particular cell lines may be attributed to the presence of different polarity compounds. Extracts **2C**, **5C**, **11C**, **4E**, **5E**, **7E** and **11E** (Figure 1) showed 40-60% of growth inhibition in MCF-7 cells.

Bacterial extracts **5C**, **1E**, **5E** and **7E** (Figure 2) demonstrated 50-70% growth inhibition against HeLa cells. Extracts **1E** and **5E** were found to be highly active against HeLa with growth inhibition of 70% and 60% respectively. Similarly, extracts **1E**, **5E**, **7E** and **9E** inhibited the growth of DU145 cells by 40% or more after 24 hr of treatment (Figure 3). Extract **1E** was the most active fraction against DU145 with about 70% growth inhibition. The comparison of data (Figures 1, 2 and 3) revealed that extract **11E** is most active in MCF-7 but comparatively has low cytotoxic activity in HeLa and DU145 cells. Also, extracts **4E** and **6E** are only active against HeLa cells (20% and 30% growth inhibition respectively) and do not affect MCF-7 and DU145 cells. Overall, the results showed that marine bacterial extracts have cell line specific cytotoxic activity.

To observe the long term effects of the bacterial extracts on the growth of cancer cell lines, the lead extracts (**2C**, **5C**, **7C, 8C, 11C, 1E, 4E, 5E, 6E, 7E, 10E** and **11E)** were further evaluated for cytotoxicity at 48 hr time point. These extracts were selected based on their activities and availability. Cell growth inhibition induced by some of the extracts was increased with time (Table 2) while for others the growth inhibition decreases slightly which may indicate the recovery of the cells, especially in the case of MCF-7 cells.

**Table 2:** The comparison of percentage growth inhibition of HeLa, MCF-7 and DU145 cells after treatment with 1 mg/mL marine bacterial extracts in triplicate for 24 and 48 hr

**Marine bacterial extract**	**HeLa**		**MCF-7**		**DU145**	
	**24 hr**	**48 hr**	**24 hr**	**48 hr**	**24 hr**	**48 hr**
2C	**44.906**	**58.003**	**61.307**	**78.608**	**30.233**	**86.682**
5C	50.000	41.931	47.173	7.654	**33.194**	**50.260**
7C	41.094	38.250	21.555	−17.160	16.597	15.263
8C	**10.151**	**23.494**	−3.180	−25.303	**−9.779**	**16.262**
11C	**39.736**	**50.176**	57.244	36.271	**−8.074**	**54.699**
1E	69.434	61.787	44.700	13.560	66.836	59.324
4E	**20.604**	**34.164**	−3.710	−10.350	**2.661**	**37.471**
5E	59.472	41.556	40.901	24.234	51.077	47.287
6E	27.547	15.256	1.502	−23.946	**−7.775**	**21.345**
7E	51.434	42.027	40.283	20.021	46.202	47.725
10E	21.925	9.215	20.936	8.992	**10.616**	**15.956**
11E	**22.453**	**29.769**	59.806	16.475	**15.371**	**45.672**

Data were normalized to DMSO treatment control.

### Apoptotic evaluations

**Figure 4:**
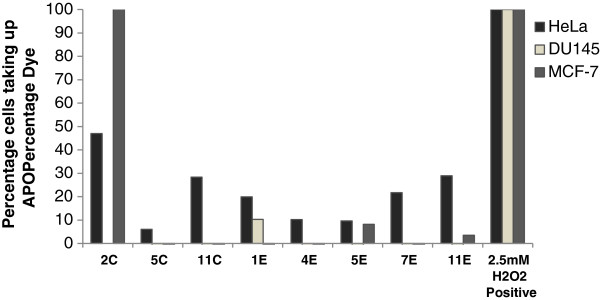
**Percent apoptosis in HeLa, DU145 and MCF-7 cells after treatment with 1 mg/mL marine bacterial extracts.** The treatment of extracts was done for 48 hr in duplicates. Data were normalized to untreated controls.

To further confirm that the mode of cell death is via apoptosis, the lead marine bacterial extracts (**2C**, **5C**, **11C, 1E, 4E, 5E, 7E, 10E** and **11E)** were evaluated by APOPercentage assay. The results for this assay showed that most of the lead extracts induced apoptosis in HeLa cells (Figure 4), while in DU145 and MCF-7 cells, no significant apoptotic effect was observed at 48 hr time point, however, the bacterial extract **2C** showed high apoptotic activity in MCF-7 cells (Figure 4).

## Discussion

The results showed that most of the microbial extracts from the brine pools of the Red Sea have displayed significant cytotoxic and apoptotic activity. Cytotoxicity which was measured by MTT assay (3-[4, 5-dimethylthiazol-2-yl]-2, 5-diphenyltetrazolium bromide), targets the activity of succinate dehydrogenase in mitochondria which in turn reduces the tetrazolium salt into formazan crystals [28]. The intensity of the color of formazan dye correlates to the number of viable cells. The isolates P1-37B (**2C**) and P3-37A (**11C** and **11E**) which has 100% sequence similarity to *Halomonas* (Table 1) showed about 60% growth inhibition (Figure 1) in MCF-7 cell line. Not much work has been done on the anticancer activities of *Halomonas* sp*.* and very few studies have been reported so far. Marine bacterial strain GWS-BW-H8hM was reported to inhibit growth of HM02 (gastric adenocarcinoma), HepG2 (hepatocellular carcinoma) and MCF7 cell lines and induce apoptosis via cell cycle arrest [29]. Highly heterogeneous polymers i.e. exopolysaccharides (EPSs) and sulphated EPSs isolated from *H. stenophila* sp. inhabiting hypersaline environment have also been reported for their pro-apoptotic effects on T-leukemia cells [30]. A range of amphiphilic siderophores, loihichelins A-F, consisting of an octapeptide comprised of D-threo-β-hydroxyaspartic acid, D-serine, L-glutamine, L-serine, L-N(δ)-acetyl-N(δ)-hydroxyornithine, dehydroamino-2-butyric acid, D-serine, and cyclic N(δ)-hydroxy-D-ornithine, appended by one of a series of fatty acids ranging from decanoic acid to tetradecanoic acid have been isolated from cultures of the marine bacterium *Halomonas* sp. LOB-5 [8]. The isolation of cytotoxic hydroxyphenylpyrrole-dicarboxylic acids i.e. 3-(4_-hydroxyphenyl)-4-phenylpyrrole-2,5-dicarboxylic acid (HPPD-1), 3,4-di-(4_-hydroxy-phenyl)pyrrole-2,5- dicarboxylic acid (HPPD-2) and the literature-known indole derivatives 3-(hydroxyacetyl)-indole, indole-3- carboxylic acid, indole-3-carboxaldehyde, and indole-3-acetic acid have also been reported from a marine *Halomonas* sp. [31]. Both HPPD-1 and HPPD-2 have shown potent antitumor- promoting activities. Two most active extracts were obtained from the isolates P1-17B (**1E**) and P5-16B (**5E**), which inhibited the cell growth by 50-70% (Figures 2 and 3) in HeLa and DU145 cells and showed sequence similarity of 100% to *Sulfitobacter* and *Halomonas* respectively (Table 1). The cytotoxic activity observed in isolates P1-37B and P3-37A (*Halomonas*) could be due to the above mentioned cytotoxic compounds from *Halomonas* sp. No published records were found in PubMed regarding the cytotoxic activities and isolation of bioactive molecules from *Sulfitobacter*. The most prominent effect was found for extract **1E** (Isolate P1-17B- *Sulfitobacter*) for HeLa and DU145 cell lines which makes it a suitable candidate for future isolation of bioactive molecules. Cytotoxic effects for the most active extracts were also observed at 48 hr time point and activity for some of these extracts were found to be lower than that at 24 hr (Table 2, see non-bold numbers). It may be due to the recovery of the cells from the effects of these extracts. In general the hydrophilic extracts were found to be more active than lipophilic extracts, which may indicate the presence of significant amount of cytotoxic compounds in the polar fractions as compared to the non-polar fractions.

To further confirm the cell death via apoptotic mode, APOPercentage assay was performed on the most active extracts. Apoptosis is a form of a programmed cell death where cell undergo specific hallmark changes during progression towards death [32]. It has been established that apoptosis plays a key role in death of cancer as well as normal cells [33]. The APOPercentage assay (http://www.biocolor.co.uk/) captures apoptotic cells representing a hallmark change on the surface of plasma membranes i.e. phosphatidylserine (PS) externalization. PS is found in the inner leaflet and is exposed to outer leaflet during apoptosis and this externalized PS acts as a recognition tag for multiple processes including macrophages and phagocytosis [34,35]. The maximum apoptotic activity was displayed by isolate P1-37B (**2C**) in both HeLa as well as MCF-7 cells (Figure 4). Similar studies targeting bacterial extracts from other marine habitats have been published previously, mainly targeting cyanobacteria [36] and bacteria associated with other marine organisms such as seaweeds and invertebrates [37] and sponges [38,39].

The cell line specific activity of the extracts may be attributed to the different polarity compounds. Previous studies on different cancer cell lines have shown that the variable response of cell lines towards apoptosis inducers may also be attributed to the cancer type i.e. biological differences in cell lines and different mechanisms of action of programmed cell death prevalent in different cancer cell lines [40]. Even the timeline of progression through apoptosis process can vary among cell lines [41,42].

## Conclusions

In conclusion, present work showed that extracts of most of the marine bacterial strains collected from interface of brine pools and sea water of the Red Sea have displayed significant growth inhibitory and apoptotic effect on the various cancer cell lines. The extracts from the isolates P1-37B and P3-37A (*Halomonas*) and P1-17B (*Sulfitobacter*) have been found to be the most potent against tested cancer cell lines and could be the lead candidates for the future work of isolation and structure elucidation of bioactive molecules.

## Abbreviations

CTD: Conductivity-temperature-depths; TFF: Tangential Flow Filtration; DMEM: Dulbecco’s Modified Eagle’s Medium; FCS: Fetal calf serum; OD: Optical density; MTT: 3-(4, 5-Dimethylthiazol-2-yl)-2, 5-diphenyltetrazolium bromide; PS: Phosphatidylserine; HeLa: Cervical carcinoma; MCF-7: Breast Adenocarcinoma; DU145: Prostate carcinoma.

## Competing interests

The authors declare that they have no competing financial interests.

## Authors’ contribution

SS and MK planned the study, wrote manuscript and performed experiments along with LE. TH isolated the strains and provides taxonomic classification of the bacterial strains. AA was responsible for the sample collection. KH was responsible for growth of the strains in large batches. US was responsible for the planning of the expedition and the cultivation experiments, provided general coordination of the study and helped in manuscript writing. VBB provided general coordination of the study and helped in manuscript writing. All authors read and approved the final manuscript.

## Pre-publication history

The pre-publication history for this paper can be accessed here:

http://www.biomedcentral.com/1472-6882/13/29/prepub
